# Optimising Non-operative Management of Inoperable Malignant Bowel Obstruction: A Clinical Review for Surgical Practice

**DOI:** 10.7759/cureus.108314

**Published:** 2026-05-05

**Authors:** Mohamed Alkashty, Ehab Kahka, Waseem Hameed, Yasser Eshra

**Affiliations:** 1 General Surgery, Wexham Park Hospital, Slough, GBR; 2 General Surgery, Ain Shams General Hospital, Cairo, EGY; 3 Colorectal Surgery, Wexham Park Hospital, Slough, GBR

**Keywords:** advanced cancer, mbo:malignant bowel obstruction, non-operative management, octreotide, palliative care

## Abstract

Inoperable malignant bowel obstruction (MBO) is a frequent and distressing complication of advanced intra-abdominal malignancy, particularly colorectal and ovarian cancer, and is associated with substantial morbidity and limited survival. Management is primarily palliative, with emphasis on symptom control rather than disease modification. For the practising surgeon, MBO represents a common clinical scenario requiring structured non-operative decision-making. This structured narrative review synthesises current evidence on non-operative management strategies for inoperable MBO, based on a systematic literature search. Interventions evaluated include pharmacological therapies, combination regimens, and selected adjunctive measures. Somatostatin analogues, particularly octreotide, demonstrate the most consistent benefit in reducing gastrointestinal secretions and vomiting. Corticosteroids and antiemetics provide adjunctive symptom control, while combination regimens appear to offer enhanced efficacy compared with monotherapy, although supporting evidence remains limited. The available literature is heterogeneous, with relatively few high-quality randomised studies and limited standardisation of outcome measures. Consequently, clinical practice is largely guided by symptom-driven, individualised approaches. Future research should prioritise well-designed multicentre trials, standardised treatment protocols, and incorporation of patient-reported outcomes to strengthen the evidence base and inform clinical practice.

## Introduction and background

Malignant bowel obstruction (MBO) is a significant complication in patients with advanced malignancy and is associated with substantial morbidity and poor prognosis. It occurs in up to 15%-28% of patients with colorectal cancer and up to 50% of those with advanced ovarian cancer [1,2]. In many cases, surgical intervention is not feasible owing to disseminated disease, poor performance status, or patient preference.

In this context, management shifts towards palliation, with the primary aim of alleviating distressing symptoms such as nausea, vomiting, abdominal pain and intolerance of oral intake. Non-operative management has therefore become a cornerstone of care in patients deemed unsuitable for surgery, incorporating pharmacological therapies, supportive measures, and selected minimally invasive interventions [3,4].

In contemporary surgical practice, MBO frequently presents in patients for whom operative intervention is not appropriate. This requires a transition from operative decision-making to structured non-operative strategies that integrate pharmacological treatment, supportive care, and multidisciplinary input. However, despite its clinical importance, pragmatic and clinically applicable frameworks to guide bedside decision-making remain limited.

This review provides a structured narrative synthesis of current evidence on non-operative management strategies for inoperable MBO. The focus is on comparative effectiveness and clinical applicability to support informed, symptom-directed management in surgical practice.

## Review

Methodology

This study was conducted as a structured narrative review. A literature search was performed using PubMed and the Cochrane Library from inception to March 2026. Search terms included combinations of “malignant bowel obstruction”, “palliative”, “octreotide”, “corticosteroids” and “antiemetics”. Reference lists of relevant articles and guidelines were also screened to identify additional studies.

Eligible studies included randomised controlled trials, observational studies, systematic reviews, and clinical guidelines addressing non-operative management of MBO. Studies were selected based on relevance to pharmacological management, supportive care strategies and clinically applicable interventions in inoperable disease. The authors performed study selection and interpretation with a focus on clinical applicability.

Given the heterogeneity in study design, patient populations, interventions and outcome measures - most of which were symptom-based-a formal meta-analysis was not feasible. Findings were, therefore, synthesised narratively, with emphasis on comparative effectiveness and clinical relevance.

This review does not follow a full Preferred Reporting Items for Systematic Reviews and Meta-Analyses (PRISMA) framework; however, key elements of systematic searching and study selection were incorporated to enhance transparency. The limitations inherent to narrative synthesis are acknowledged.

Overview of non-operative management

The principal non-operative treatment modalities for inoperable MBO are summarised in Table 1, highlighting their clinical benefits, limitations and overall strength of evidence.

**Table 1:** Summary of principal non-operative treatment modalities for Inoperable malignant bowel obstruction. SBO, small bowel obstruction

Treatment modality	Evidence strength	Key benefits	Key risks/limitations	Notes
Octreotide	High	Rapid reduction in gastrointestinal secretions, vomiting, and nasogastric output	Generally well tolerated; limited impact on overall quality of life	First-line pharmacological option; superior to hyoscine butylbromide for rapid symptom control
Dexamethasone (corticosteroids)	Moderate	Reduces bowel wall oedema and nausea; useful in combination regimens	Hyperglycaemia, proximal myopathy, adrenal suppression; variable response	Best used as adjunctive therapy rather than monotherapy
Metoclopramide	Moderate	Antiemetic and prokinetic effects; improves symptoms in partial obstruction	May worsen colicky pain in complete obstruction; extrapyramidal effects	Appropriate mainly in incomplete obstruction
Olanzapine	Low-moderate	Alternative antiemetic for refractory nausea and vomiting	Sedation; limited comparative evidence	Consider when conventional antiemetics are ineffective or poorly tolerated
Combination therapy (e.g. octreotide + dexamethasone + metoclopramide)	Moderate	Synergistic symptom control; often faster improvement than monotherapy	Evidence largely from small observational studies	Increasingly used in practice; further high-quality studies required
Parenteral nutrition	Low–moderate	Nutritional support in selected patients with a reasonable prognosis	Infection, line complications, metabolic burden	Individualised use; not primarily for symptom control
Gastrografin (water-soluble contrast)	Low–moderate	Potential therapeutic effect in partial obstruction by drawing fluid into the bowel lumen	Risk of aspiration; limited evidence in malignant obstruction compared with adhesive SBO	Selective use in partial obstruction, with caution in frail patients
Colonic stenting	moderate	Effective palliation may avoid surgery and reduce hospital stay	Migration, re-obstruction, perforation	Minimally invasive option in selected large bowel obstructions

Somatostatin analogues

Somatostatin analogues, particularly octreotide, are among the most extensively studied pharmacological interventions in MBO. Their mechanism of action includes inhibition of gastrointestinal secretions, reduction of splanchnic blood flow, and decreased intestinal motility, resulting in reduced intraluminal pressure [5].

Randomised and prospective studies have demonstrated that octreotide reduces the frequency of vomiting and nasogastric output compared with placebo and anticholinergic agents such as hyoscine butylbromide [6,7]. Reported rates of symptom improvement range from 60% to 90%, with a generally favourable safety profile [8].

Current Multinational Association of Supportive Care in Cancer (MASCC) guidelines support the use of octreotide as first-line pharmacological therapy in patients with inoperable MBO [9].

Corticosteroids

Corticosteroids, most commonly dexamethasone, are widely used as adjunctive therapy in MBO. Their proposed mechanism involves reducing bowel wall oedema and exerting anti-inflammatory effects, which may contribute to partial relief of obstruction [10].

The evidence supporting corticosteroid use is variable. Some studies report symptom improvement, whereas others demonstrate inconsistent or limited benefit [11]. Adverse effects, including hyperglycaemia, proximal myopathy, and adrenal suppression, are relatively common and may limit prolonged use [12].

Despite these limitations, corticosteroids remain an important component of combination regimens, where they may enhance overall symptom control.

Antiemetics and prokinetic agents

Antiemetic therapy is central to symptom control in MBO. Agents such as haloperidol and levomepromazine are commonly used for refractory nausea and vomiting [13].

Metoclopramide, a prokinetic agent, has a dual role in enhancing gastric emptying and reducing nausea. However, its use is generally limited to partial obstruction, as it may exacerbate colicky pain with complete obstruction [14].

Olanzapine has emerged as an alternative antiemetic, with small studies suggesting favourable efficacy and tolerability, although robust comparative data remain limited [15].

Combination therapy

Combination pharmacological therapy is increasingly adopted in clinical practice. The most commonly described regimen includes octreotide, dexamethasone, and metoclopramide.

Observational studies and small clinical trials suggest that this *triple therapy* approach may provide faster and more complete symptom relief compared with monotherapy, often within one to five days [16,17].

However, high-quality comparative evidence remains limited. The rationale for combination therapy lies in the synergistic targeting of multiple pathophysiological mechanisms, supporting its pragmatic use in clinical practice.

Adjunctive and supportive measures

Supportive care remains fundamental in the management of MBO. Fluid and electrolyte correction is essential, particularly in patients with significant vomiting and dehydration [18].

Nasogastric decompression may provide temporary symptom relief but is often poorly tolerated over prolonged periods. Venting gastrostomy can be considered in selected patients to achieve more durable decompression [19].

The role of parenteral nutrition remains controversial and should be individualised, typically reserved for carefully selected patients with reasonable prognosis and functional status [18,19].

Water-soluble contrast agents such as Gastrografin have an established role in adhesive small-bowel obstruction; however, their role in malignant small-bowel obstruction remains uncertain. A Cochrane review identified only one small feasibility study and concluded that there is insufficient evidence to support a diagnostic or therapeutic benefit in this setting [20]. This is reflected in MASCC guidelines, which do not recommend routine use due to limited evidence (level V, grade D) [21]. In clinical practice, Gastrografin may be considered selectively in patients with suspected partial obstruction and low aspiration risk, but should be regarded as an adjunct rather than standard care.

Endoscopic and minimally invasive interventions

Endoscopic stenting represents an important adjunct in selected patients, particularly in malignant large bowel obstruction. It can provide effective symptom relief and may avoid the need for surgery in patients with limited life expectancy [22].

However, stenting is not suitable for all patients and is associated with complications, including migration, re-obstruction, and perforation. Its use should therefore be considered within a multidisciplinary framework, taking into account disease extent, anatomical factors, and patient goals of care.

Comparative effectiveness and safety

Comparative studies suggest that octreotide is more effective than anticholinergic agents such as hyoscine butylbromide in achieving rapid symptom control [6,7].

Combination therapy may offer enhanced efficacy compared with single-agent treatment; however, the supporting evidence is largely derived from small studies [16,17].

Overall, non-operative management is generally well tolerated. Octreotide has a favourable safety profile, whereas corticosteroids are associated with a higher risk of adverse effects, particularly with prolonged use [12].

Patient outcomes and quality of life

Symptom control remains the primary goal in the management of MBO. Pharmacological therapy is effective in reducing nausea, vomiting, and pain, although improvements in overall quality of life are generally modest [23].

Median survival in patients with inoperable MBO is limited, often less than two months, and medical management does not significantly influence survival outcomes [24].

Importantly, non-operative management is associated with shorter hospital stays and an increased likelihood of discharge to home or hospice care, supporting its role in patient-centred palliative care [25].

Commonly used pharmacological regimens and dosing ranges are summarised in Table 2. These reflect typical clinical practice and guideline-informed use, recognising that high-quality evidence to define optimal dosing remains limited and that treatment should be individualised.

**Table 2:** Commonly used pharmacological regimens in inoperable malignant bowel obstruction. SC, subcutaneous; IV, intravenous; TDS, three times daily; BD, twice daily

Medication	Typical dosing range	Route	Key considerations
Octreotide	100-300 mcg/24 hours (continuous infusion) or 50-100 mcg SC TDS	SC/IV infusion	First-line therapy; reduces gastrointestinal secretions and vomiting; can be titrated based on response
Dexamethasone	4-16 mg daily	IV/SC/oral	Adjunctive therapy; reduces bowel wall oedema; consider a short course and taper if prolonged
Metoclopramide	10 mg TDS (up to 60 mg/day)	SC/IV/oral	Prokinetic and antiemetic; avoid in complete obstruction
Haloperidol	0.5-1.5 mg BD-TDS	SC/oral	Effective antiemetic for refractory nausea and vomiting
Levomepromazine	6.25-12.5 mg nocte (up to 25-50 mg/day)	SC/oral	Broad-spectrum antiemetic; sedating; useful in advanced disease
Hyoscine butylbromide	20 mg SC every 4-6 hours or 60-120 mg/24 h infusion	SC/IV infusion	Antisecretory and antispasmodic; less effective than octreotide for secretion control
Olanzapine	2.5-5 mg nocte	Oral	Alternative antiemetic; useful when standard agents are insufficient

Discussion

**Figure 1:**
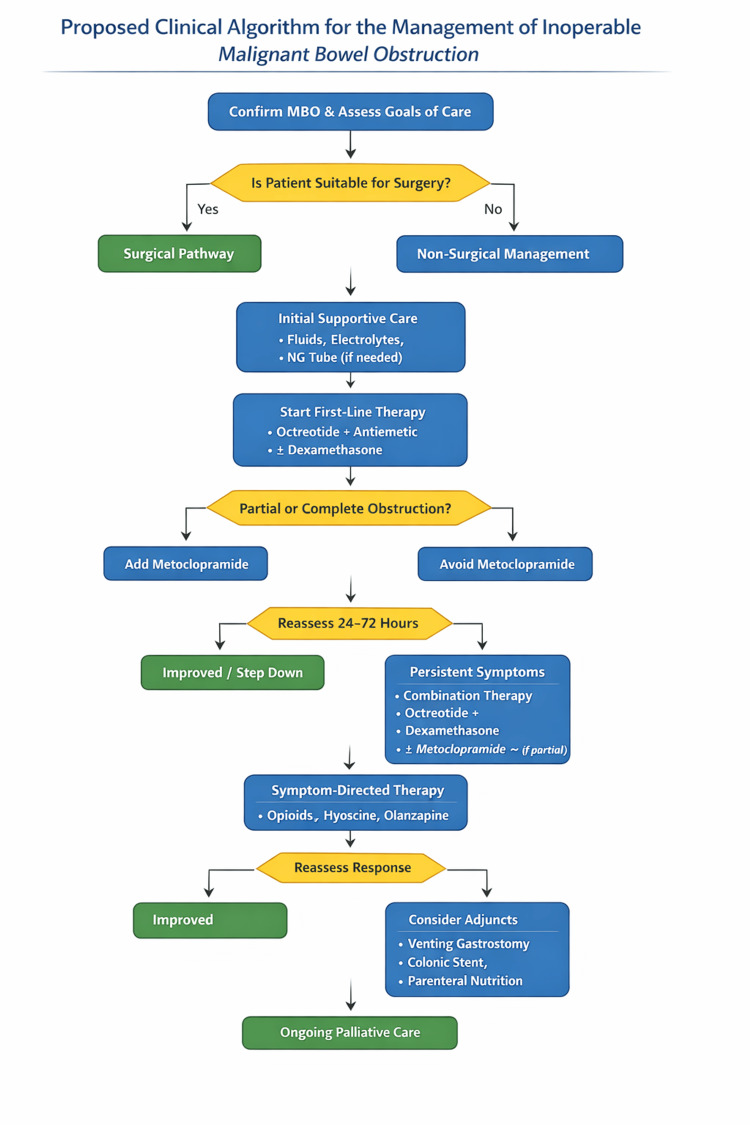
Proposed clinical algorithm for the management of inoperable malignant bowel obstruction. Image created by authors. MBO, malignant bowel obstruction; NG, nasogastric

A pragmatic, symptom-driven clinical algorithm for managing inoperable MBO is shown in Figure 1.

For the practising surgeon, the management of inoperable MBO extends beyond the decision not to operate, requiring a structured and proactive non-operative approach. The proposed algorithm integrates the available evidence into a pragmatic framework to support bedside clinical decision-making.

This review highlights that non-operative management remains the cornerstone of care in inoperable MBO. Among available therapies, octreotide has the most consistent evidence of efficacy, particularly in reducing gastrointestinal secretions and vomiting, as supported by randomised and prospective studies and reflected in MASCC guideline recommendations [6-9,21].

Current practice is largely guided by pragmatic, symptom-driven titration rather than standardised protocols, reflecting the heterogeneity of the evidence base and variability in clinical presentation [3,4]. This underscores the evolving role of the surgeon in advanced malignancy, where management increasingly involves integration of operative judgement with evidence-informed non-operative strategies.

Management of inoperable MBO is inherently multidisciplinary, requiring close collaboration between surgeons, palliative care teams, and oncologists to optimise symptom control and align treatment with patient goals [1,25].

Combination regimens incorporating corticosteroids and antiemetics may provide improved symptom control, consistent with the multifactorial pathophysiology of MBO. However, the supporting evidence remains limited and heterogeneous, largely derived from small observational studies, a limitation also reflected in current guideline recommendations [16,17,21].

The findings of this review should be interpreted in the context of its design as a structured narrative synthesis. Although efforts were made to include relevant and contemporary literature, the absence of formal quantitative analysis limits the strength of comparative conclusions.

Future research should prioritise well-designed multi-centre randomised studies with standardised, patient-centred outcome measures, including quality of life and symptom burden, to strengthen the evidence base and inform clinical practice.

## Conclusions

For the practising surgeon, effective non-operative management of inoperable malignant bowel obstruction is central to achieving symptom control and delivering patient-centred care. Somatostatin analogues, particularly octreotide, represent the most consistently supported pharmacological therapy, with adjunctive roles for corticosteroids and antiemetics. Combination regimens may offer enhanced symptom control; however, the supporting evidence remains limited and heterogeneous. Further high-quality research is required to optimise treatment strategies, standardise care, and better define clinically meaningful outcomes.
